# Puumala Hantavirus-Induced Hemorrhagic Fever with Renal Syndrome Must Be Considered across the Borders of Nephrology to Avoid Unnecessary Diagnostic Procedures

**DOI:** 10.1371/journal.pone.0144622

**Published:** 2015-12-09

**Authors:** Daniel Kitterer, Stephan Segerer, M. Dominik Alscher, Niko Braun, Joerg Latus

**Affiliations:** 1 Department of Internal Medicine, Division of General Medicine and Nephrology, Robert-Bosch-Hospital, Stuttgart, Germany; 2 Division of Nephrology, University Hospital, Zurich, Switzerland; 3 Nephrology Center, Stuttgart, Germany; University of Texas Medical Branch, UNITED STATES

## Abstract

**Background:**

Nephropathia epidemica (NE), a milder form of hemorrhagic fever with renal syndrome, is caused by Puumala virus and is characterized by acute kidney injury and thrombocytopenia.

**Methods:**

A cross-sectional prospective survey of 456 adult patients with serologically confirmed NE was performed.

**Results:**

Of the 456 investigated patients, 335 had received inpatient treatment. At time of admission to hospital, 72% of the patients had still an AKI and thrombocytopenia was present in 64% of the patients. The 335 patients were treated in 29 different hospitals and 6 of which had nephrology departments. 10 out of 335 patients received treatment in university hospitals and 63% of patients admitted themselves to hospital. Initially, the patients were admitted to 12 different clinical departments (29% of the patients were referred to a nephrology department) and during the course of the disease, 8% of the patients were transferred to another department in the same hospital and 3% were transferred to a nephrology department at another hospital. Regarding diagnostic procedures, in 28% of the inpatients computed tomography to exclude pulmonary embolism or due to severe gastrointestinal symptoms, lumbar puncture to exclude meningitis, magnetic resonance tomography of the brain owing to suspected stroke because of visual disorders, gastroscopy, or colonoscopy due to gastrointestinal symptoms was performed at time of admission to hospital.

**Conclusions:**

NE must be considered by physicians across the borders of nephrology to avoid unnecessary diagnostic procedures especially in areas where NE is endemic.

## Introduction

Hantaviruses are emerging viruses causing hemorrhagic fever with renal syndrome (HFRS) in Asia and Europe (Old World hantaviruses) [1–3]. In the Americas, infection by New World hantaviruses might lead to hantavirus cardiopulmonary syndrome (HCPS), with case fatality rates of up to 35% [4]. In fact, there is a growing list of affected countries (developing and developed countries), which is leading to public health concerns [5, 6]. Both HFRS and HCPS are associated with acute thrombocytopenia and changes in vascular permeability, and both syndromes can include renal and pulmonary symptoms [3]. The most common causative agent for HFRS in Germany is Puumala virus [5]. Puumala virus causes nephropathia epidemica (NE), which is a milder form of HFRS [7, 8], and patients typically present with acute kidney injury and thrombocytopenia. At the beginning of the disease, patients typically present with flu-like symptoms (fever, headache, nausea or vomiting, or visual disturbances). Furthermore, a high percentage of patients with Puumala virus infection present with severe gastrointestinal symptoms, e.g. abdominal pain, nausea and vomiting [8–14]. Laboratory investigations at the beginning of NE reveal that a high proportion of patients show signs of systemic inflammation with leukocytosis and elevated levels of C-reactive protein and procalcitonin [9, 14–18]. Therefore, the differential diagnosis is broad, including, for example, leptospirosis (Weil’s disease, Stuttgart disease), sepsis, autoimmune disease, and thrombotic microangiopathy in patients with AKI and thrombocytopenia or other viral or bacterial diseases in patients presenting with e.g. fever, headache and elevated inflammatory markers

This might lead the physicians to perform various diagnostic procedures at time of admission to hospital and due to the wide range of clinical signs and symptoms patients might be admitted to different clinical departments. Until now, there are no reports about performed procedures during work-up of patients with NE after admission to hospital and no data about the involved medical fields. Therefore, we analyzed the diagnostic procedures that were undertaken in a large cohort of patients with acute NE in an endemic area of Southern Germany after admission to hospital and studied the different clinical departments that were involved in treatment of patients with acute NE.

## Materials and Methods

### Patients

Between 2001 and 2012, 7,476 patients with serologically and clinically confirmed NE were reported to the Robert Koch Institute in Berlin (Robert Koch Institute, SurvStat, www.3.rki.de/SurvStat). In cooperation with four selected local health authorities in southern Germany (Stuttgart, Böblingen/Sindelfingen, Esslingen, Reutlingen), all infected patients between 2001 and 2012 were contacted via mail requesting that they make an appointment in our outpatient clinic (in total, 1,570 patients were serologically confirmed to have hantavirus infection).

Between September 2012 and April 2013, 456 out of the 1,570 contacted patients with serologically and clinically confirmed NE were included in our study. All patients gave written consent before participating in the study, which was approved by the Ethics Committee of the Ethics Commission of the State Chamber of Medicine in Baden-Württemberg (Stuttgart) (F-2012-046). Studies were conducted in concordance with the Declaration of Helsinki.

### Data acquisition

#### Acute phase of NE

Clinical and laboratory data at the time of diagnosis and during the acute course of the disease were obtained from medical reports and files from each patient. Patients who were treated as outpatients by general practitioners or nephrologists were excluded from the analysis.

## Results

Of the 456 investigated patients, 335 had received inpatient treatment, and 121 had received outpatient treatment by general practitioners or nephrologists. The baseline characteristics of the study population treated as inpatients are shown in Table 1. At time of admission to hospital, 72% of the patients had still an AKI and thrombocytopenia was present in 64% of the patients. The course of the disease and the diagnostic procedures used are shown in Table 2. The 335 inpatients were treated in 29 different hospitals: 10 patients received treatment in university hospitals, and six of the hospitals had nephrology departments. Approximately 63% of patients admitted themselves to hospital. Initially, the patients were admitted to 12 different clinical departments (29% of the patients were referred to a nephrology department). During the course of the disease, 8% of the patients were transferred to another department in the same hospital and 3% were transferred to a nephrology department at another hospital. Consultation during hospital stay was carried out in 10% of the patients (mostly nephrologists, ophthalmologists, neurologists, and urologists).

**Table 1:** Baseline characteristics of study population during acute hantavirus infection; CRP C-reactive protein; LDH Lactate dehydrogenase; # Clinical signs and symptoms and laboratory values were taken at time of admission to hospital.

Variable	Inpatients (n = 335)
Female/male	120/215
Age at diagnosis (years)	47 (40–59)
**Symptoms/clinical signs#**	
Pain of the limbs	69%
Headache	68%
Back pain	68%
Nausea/Vomiting	46%
Abdominal pain	31%
Visual disorders	23%
Diarrhea	21%
Fever	90%
**Laboratory findings #**	
Creatinine (mg/dl [0.5–1.4])	1.9 (1.2–3.6)
Highest measured Creatinine (mg/dl [0.5–1.4])	2.9 (1.7–4.9)
Platelets (10^9^/L [>150])	123 (84–200)
CrP (mg/dl [0.1–0.4])	4 (2.4–6.8)
Elevated LDH	65%
Urinary analysis #	
• Proteinuria	80%
• Hematuria	74%
• Leukocyturia	12%

**Table 2:** Course of the disease in 335 NE patients treated in-hospital; CT computed tomography; RRT Renal replacement therapy.

Variable	Inpatients (n = 335)
Referred to hospital by general physician	123
Self-admittances to hospital	212
Initially admitted to	
• General Internal medicine department	171
• Nephrology department	96
• Neurology department	7
• Urology department	13
• Gastroenterology department	21
• Cardiology department	10
• Hematology and oncology department	8
• Surgical department	3
• Ear, nose, and throat department	1
• Pediatric department	2
• Gynecology department	1
• Intensive care unit	2
Transferred intra-hospital	26
• Urology to nephrology	8
• Neurology to nephrology	6
• General Internal medicine to nephrology	6
• Surgical to nephrology	2
• Others	4
Transferred to another hospital (all nephrology department)	10
Initiation of RRT	5
Diagnostic procedures during course of the disease	
• CT diagnostic with contrast medium	17
• Cranial CT scan without contrast medium	22
• Lumbar puncture	17
• Magnetic resonance tomography	20
• Gastroscopy	18
• Colonoscopy	5
• Endosonography	1
• Bronchoscopy	1
• Kidney biopsy	1
• Diagnostic of suspected autoimmune disease	70
• Abdominal ultrasound	278
• Chest-x-ray	206

Regarding diagnostic procedures that were undertaken in the group of inpatients during the acute course of the disease, in 28% of the patients, CT scan, lumbar puncture, magnetic resonance tomography (MRI) of the brain, gastroscopy, or colonoscopy was done. Within our study population, seven patients were initially admitted to a neurology department. In five out of seven patients, lumbar puncture was performed, owing to suspected meningitis, revealing negative results in any of the patients. In 6% of the patients admitted to hospital, CT with contrast media was performed. Computed tomography was mainly undertaken to exclude pulmonary embolism in patients with elevated D-dimer at the time of admission to hospital and in 12 patients, CT of the abdomen and pelvis was done because the patients had severe gastrointestinal symptoms.

During diagnostic work-up of AKI at the time of admission to hospital, laboratory investigation for suspected autoimmune disease was performed in 21% of the patients. During the acute course of the disease, 4% of the inpatients received treatment at an intensive care unit and ten patients were treated with intravenous steroids during NE.

## Discussion

Differential diagnosis of acute kidney injury with signs of systemic inflammation is broad, including, for example, sepsis and thrombotic microangiopathy. Until now, there have been no reports about diagnostic procedures performed in the differential diagnosis of patients presenting mostly with systemic inflammation, including fever, elevated inflammatory markers, and acute kidney injury with thrombocytopenia in an area where NE is endemic

In our study population, 97 invasive diagnostic procedures (e.g., CT, lumbar puncture, or endoscopy) in 335 patients were performed (in the absence of the number of chest X-rays and abdominal ultrasound scans, see Table 2). Remarkably, 12 different disciplines of medicine were involved at first contact with the patients, and diagnostic procedures performed at time of admission to hospital varied with regard to the medical discipline with which the patients had first contact. A nephrology department was present in only 29% of the hospitals involved, which indicates the importance of knowledge of the disease across the boarders of nephrology. In our study population, seven patients were initially admitted to a neurology department because of fever, visual disorders, and headache. In five out of seven patients, lumbar puncture was performed to exclude meningitis, whereas endoscopy (because of gastrointestinal symptoms, mostly abdominal pain) was more often performed in patients admitted to a gastroenterology department or to a general internal medicine department in which the head of department was a gastroenterologist. Invasive diagnostic procedures might lead to major complications, e.g. bleeding complications, especially in patients with NE, because thrombocytopenia was present in 65% of the patients at the time of admission to hospital.

Computed tomography was mainly undertaken to exclude pulmonary embolism in patients with elevated D-dimer at the time of admission to hospital. None of the patients had pulmonary embolism. In a small proportion of patients, only mild pleural effusions and no pulmonary edema were present according to CT results. Computed tomography is associated with radiation exposure and the use of contrast medium might aggravate severity of acute kidney injury (71% of patients had acute kidney injury at the time that CT with contrast medium was carried out). In 2010, Laine *et al*. [19] reported decreased levels of natural anticoagulants, shortened thrombin time, and enhanced fibrinolysis in patients with acute NE. Therefore, elevated D-dimer could be expected at the time of diagnosis of NE.

Laboratory investigations due to suspected autoimmune disease were performed more frequently in patients admitted to a nephrology department; this raises public health costs and might cause false positive results with ensuing implications for the patient (e.g. kidney biopsy due to suspected glomerulonephritis).

Remarkably, acute leptospirosis was considered as a differential diagnosis in only a few patients, although acute leptospirosis and acute hantavirus infection share many clinical manifestations and certain epidemiological features [20]. Very recently, we could show that procalcitonin levels were elevated in 56% of patients with NE, which indicates that procalcitonin might not be useful in differentiating hantavirus infection from bacterial infection [18].

Our study design has several limitations that have to be addressed. Only one-third of NE occurs with typical clinical signs, resulting in extensive under-reporting, especially in younger patients with mild disease. In our study, patients were contacted by mail and asked to attend the outpatient clinic for follow-up investigations, which might lead to a selection bias because more patients with a more severe course of the disease might have been included in the study. In addition, regarding the acute course of the disease, we retrospectively studied medical case reports, which are associated with various limitations.

In conclusion, NE must be considered by physicians across the borders of nephrology to avoid unnecessary diagnostic procedures especially in areas where NE is endemic. Serologically, confirmation of NE takes only several hours and, therefore, the use of particularly invasive diagnostic procedures, except abdominal ultrasound to exclude post-renal acute kidney injury, should be extremely limited.
